# Authors’ Response to Peer Reviews of “Use of a Specialist Telephone Consultation Line for Long COVID in Primary Care in British Columbia: Retrospective Descriptive Quality Improvement Study”

**DOI:** 10.2196/89710

**Published:** 2026-02-10

**Authors:** Saniya Kaushal, Jastinder Bhandal, Peter Birks, Jesse Greiner, Adeera Levin, Michelle Malbeuf, Zachary Schwartz

**Affiliations:** 1 Providence Health Care Research Institute and Provincial Health Services Authority, 1081 Burrard Street, Vancouver, BC, V6Z 1Y6, Canada, 1 6047511478; 2 Postgraduate Medical Education – Internal Medicine, Toronto Metropolitan University, Toronto, ON, Canada; 3 School of Medicine, University of Limerick, Limerick, Ireland; 4 Faculty of Medicine, The University of British Columbia, Vancouver, BC, Canada

**Keywords:** internal medicine, long COVID, COVID-19, SARS-CoV-2, GP, general practice, general practitioner, consult, respiratory, infectious, respiration, primary care, telephone, telehealth

*This is the authors’ response to peer-review reports for “Use of a Specialist Telephone Consultation Line for Long COVID in Primary Care in British Columbia: Retrospective Descriptive Quality Improvement Study.”*

## Round 1 Review

### Reviewer AG [1]

#### Major Comments

*1. The authors of this study* [2] *mention that 6 calls were excluded but never gave an analysis of the trend of the calls.*

**Response:** We now specify the reasons for excluding the 6 calls, which included unclear documentation, no discernible COVID-related question, or insufficient information. These exclusions are described in the Data Source and Call Selection subsection of the Methods. For transparency, we also clarify that excluded calls were logged to ensure consistent application of inclusion criteria.

*2*. *Can the 6 calls drive some conclusions that can assist with the paper?*

**Response:** As the 6 excluded calls lacked sufficient information to categorize meaningfully, their content was not analyzed to avoid introducing misclassification bias. This has been noted as a limitation in the Discussion, where we suggest that future audits could include minimal call documentation to allow sensitivity analyses.

*3*. *Can the author give a trend line for the period of these calls and indicate if there are related cases among different calls?*

**Response:** Temporal trends in call volume and related case patterns across pandemic phases and relative to vaccine rollout are now presented in the Results. Figure 1 illustrates these changes over time.

### Anonymous [3]

#### Major Comments

*1*. *The study design was relatively simple, with only age and gender collected for basic characteristics and no mention of past medical history, which had a greater impact on the study results, especially since the study results showed a high rate of reported respiratory symptoms. In addition, the 40‐49 year age group also had a high prevalence of chronic respiratory illnesses; previous respiratory illnesses are bound to worsen to varying degrees after a COVID infection. Despite the high probability of missing visits or ambiguous data, the collection of past medical history is something that I personally feel should have been added, and missing data need to be accounted for.*

**Response:** We agree that the lack of past medical history is a limitation inherent to the service-level documentation available for this quality improvement project. We have now added this explicitly to the Limitations section and suggested that future Rapid Access to Consultative Expertise (RACE) audits include optional fields for past medical history and data completeness tracking to improve interpretability (Study Strengths and Limitations section of the Discussion).

*2*. *As a quality improvement study, I believe that the original COVID–general internal medicine–Post-Infection Care RACE line should be introduced (such as through flowcharts) to identify problems in the follow-up process and problems affecting the results of the study and to propose more specific improvement measures such as special training for follow-up personnel to guide the enrolled patients to more accurately provide the information needed for the study.*

**Response:** The Methods section now includes a concise description of the original RACE line consultation process, outlining call initiation, triage by specialists, and documentation back to primary care providers.

We also clarify how the service supports primary care provider decision-making and identify potential improvement measures, including educational resources and standardized clinical algorithms, to guide future quality improvement initiatives (Methods and Discussion sections).

*3*. *There are too many confounding factors affecting the results, and the author team does not seem to have mentioned measures to minimize the impact of confounding factors on the results of the study.*

**Response:** We expanded the Limitations to address key confounding factors, such as regional variation in access or awareness of the RACE line, heterogeneity in documentation quality, and evolving case definitions of long COVID. We also note that future evaluations could incorporate structured data fields and prospective data collection to reduce confounding and enhance data reliability (Study Strengths and Limitations section in the Discussion).

## Abbreviations

RACE: Rapid Access to Consultative Expertise
